# Nanosponges for Drug Delivery and Cancer Therapy: Recent Advances

**DOI:** 10.3390/nano12142440

**Published:** 2022-07-16

**Authors:** Siavash Iravani, Rajender S. Varma

**Affiliations:** 1 Faculty of Pharmacy and Pharmaceutical Sciences, Isfahan University of Medical Sciences, 81746-73461 Isfahan, Iran; 2 Regional Centre of Advanced Technologies and Materials, Czech Advanced Technology and Research Institute, Palacký University in Olomouc, Šlechtitelů 27, 783 71 Olomouc, Czech Republic

**Keywords:** nanosponges, cyclodextrin-based nanosponges, DNAzyme nanosponges, ethylcellulose nanosponges, drug delivery, cancer therapy

## Abstract

Nanosponges with three-dimensional (3D) porous structures, narrow size distribution, and high entrapment efficiency are widely engineered for cancer therapy and drug delivery purposes. They protect the molecular agents from degradation and help to improve the solubility of lipophilic therapeutic agents/drugs with targeted delivery options in addition to being magnetized to attain suitable magnetic features. Nanosponge-based delivery systems have been applied for cancer therapy with high specificity, biocompatibility, degradability, and prolonged release behavior. In this context, the drug loading within nanosponges is influenced by the crystallization degree. Notably, 3D printing technologies can be applied for the development of novel nanosponge-based systems for biomedical applications. The impacts of polymers, cross-linkers, type of drugs, temperature, loading and mechanism of drug release, fabrication methods, and substitution degree ought to be analytically evaluated. Eco-friendly techniques for the manufacturing of nanosponges still need to be uncovered in addition to the existing methods, such as solvent techniques, ultrasound-assisted preparation, melting strategies, and emulsion solvent diffusion methods. Herein, the recent advancements associated with the drug delivery and cancer therapy potential of nanosponges (chiefly, cyclodextrin-based, DNAzyme, and ethylcellulose nanosponges) are deliberated, focusing on the important challenges and future perspectives.

## 1. Introduction

**Figure 1:**
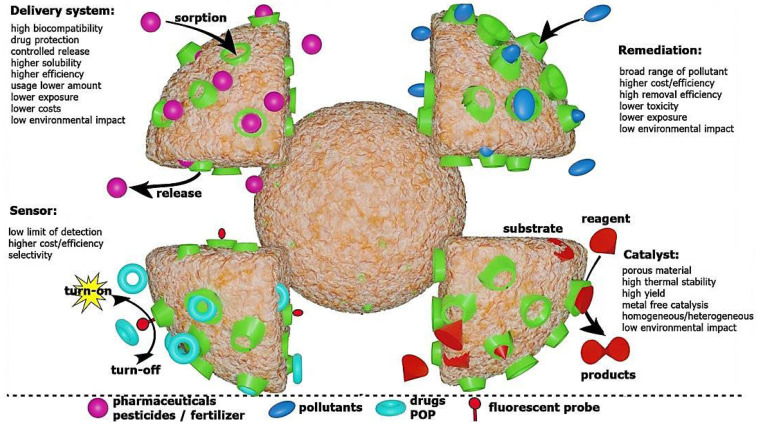
Cyclodextrin-based nanosponges with their advantages and applications. Adapted from [12] with permission. Copyright 2022 Frontiers (CC BY 4.0).

Nanomaterials have been widely explored as potential drug delivery and cancer therapy agents; however, detailed studies are still required to find efficient delivery systems with high biocompatibility and biodegradability properties for specific and targeted therapy [1,2,3,4]. Additionally, the interactions of these nanomaterials with physiological environments, along with their nanotoxicological assessments, pharmacokinetic/pharmacodynamic issues, metabolism, and drug release mechanisms (e.g., diffusion, erosion, targeting, swelling, dissolution, and osmosis), still need to be comprehensively addressed, especially for clinical translation studies [4,5,6,7,8,9]. Among these created nanomaterials, nanosponges with suitable penetration, absorption, biocompatibility, bioavailability, and stability have been studied for targeted and sustained drug delivery and cancer therapy [10]. Nanosponges are solid, crosslinked, polymeric, nano-sized, porous structures; these can be defined as hydrophilic, water-insoluble, and supramolecular three-dimensional (3D) hyper-reticulated nanoporous structures, exhibiting significant stability over a wide range of temperatures and pH levels. They have shown attractive benefits, such as high biocompatibility, biodegradability, and low cytotoxicity, making them suitable for biomedical applications [11]. For instance, cyclodextrin-based nanosponges have excellent potential for the production of inclusion and non-inclusion complexes with a variety of drugs/active molecules, offering an effective therapeutic vehicle for transferring drugs with low bioavailability. In inclusion complex nanosponges, the drug forms an inclusion complex with the cyclodextrin molecule. However, in non-inclusive complex nanosponges, the drug molecule becomes entrapped or imbibed into their porous nanostructures. Among nanosponges, cyclodextrin-based structures have been widely explored for drug delivery, remediation, sensing, and catalytic applications (Figure 1) [12]. The cavity of nanosponges permits the addition of hydrophobic molecules, and the more hydrophilic outer polymeric network enables it to accommodate less lipophilic molecules. It has been revealed that the solubility/degradability of the drugs can be improved when they are incorporated into the nanosponges, thus enhancing their bioavailability. As an example, nanosponges have been formulated to enhance the solubility and bioavailability and to reduce the oral dose of the anticancer drug, lapatinib [13]. On the other hand, one of the disadvantages of these nanostructures is their ability to include only small molecules. Indeed, the application of nanosponges is still at an early stage, and future explorations ought to focus on designing innovative nanosponge-based systems (either alone or in the presence of other polymers) with potential pharmaceutical and biomedical applications [12,14,15,16]. An assortment of nanosponges has been created, namely titanium-based, metallic, *β*-cyclodextrin, hyper-crosslinked polystyrene, silicon-based, DNAzyme, and ethylcellulose nanosponges [17]. The mesh-like/colloidal structures of nanosponges make them ideal candidates for the encapsulation of different compounds, including drugs, phytochemicals, volatile oils, antineoplastic agents, genetic substances, and proteins/peptides, among others [18,19,20]. As an example, peptide nanosponges (~80 nm) were designed for cell-based cancer therapy [19]. Furthermore, cyclodextrin-based nanosponges were introduced as effective and safer alternatives for removing toxic molecules such as indole in the body, reducing the dialysis frequency and avoiding the hepatic and cardiac toxicity [21].

Nanosponges (~200–300 nm) exist in both crystalline and para-crystalline forms, which mostly depend on the reaction/synthesis and processing conditions. The property pertaining to the crystallization of nanosponges can help in controlling and governing their drug-loading capacity [17]. Several techniques have been introduced for the synthesis of nanosponges, such as the interfacial phenomenon, hot melting process, hyper-crosslinked cyclodextrin, ultrasound-assisted, solvent condensation, microwave (MW)-assisted synthesis, interfacial condensation, mechanochemical synthesis, chain-growth poly-condensation, and emulsion solvent evaporation methods [22]. Notably, greener approaches with safety, cost-effectiveness, and eco-friendliness for the synthesis of nanosponges still need to be attended to [23,24,25,26,27]. For instance, the MW synthesis technique was deployed to synthesize crystalline cyclodextrin nanosponges with a narrow particle size distribution via the reaction of cyclodextrin with suitable cross-linkers in polar aprotic solvents (e.g., dimethylformamide) [28]. In addition, the ultrasonic-assisted synthesis of nanosponges with uniform spherical sizes was reported, with the benefits of solvent-free processes and environmentally benign features [12,29]. Widely explored nanosponges such as the cyclodextrin-based nanosponges have been synthesized under different types of stimuli-responsive methods that allow the acquisition of molecularly imprinted, plain, and modified nanosponges [30]. For instance, cyclodextrin nanosponges obtained via the molecularly imprinted approaches have shown remarkable selectivity/specificity towards molecular agents, making them suitable for various biomedical applications [31]. Cyclodextrin nanosponges have presented the alluring benefits of unique architectures, a high crosslinked 3D network, negligible toxicity, sustainability, environmental friendliness, low cost, and hosting potentials for a variety of molecular agents, which renders them suitable for the fields of bio- and nanomedicine [32]. The formation of assorted complexes of nanosponges with hydrophilic or lipophilic molecules has been explored for targeted delivery, along with their protection from degradability [16,22,33].

The surface functionalization/modification of these nanosponges can be performed by applying carbon nanotubes, silver nanowires, and titanium dioxide (TiO_2_), among others [34,35]. As an example, the cyclodextrin-based nanosponges were surface functionalized with cholesterol; these are dispersible in the cells and are responsible for different protein bindings or cell interactions. After that, doxorubicin was loaded into the functionalized nanosponges to improve the bioavailability and targeted release of this anticancer drug [36]. Indeed, several studies have shown the excellent potential of nanosponges with their special porous structures for entrapping or encapsulating drugs/therapeutic agents, thereby reducing their toxicity and possible side effects [37]. They have also shown a programmable and sustained release of drugs compared to the conventional drug delivery systems [38]. In one study, chitosan nanosponges were designed for improving the drug penetration through skin, with no noticeable toxicity. Accordingly, the skin permeation was enhanced compared to the free model drug, providing efficient trans-epidermal drug delivery [39]. There are still some crucial factors that should be systematically evaluated, particularly in the clinical translation studies of nanosponge-based dosage forms, encompassing pharmacokinetics, recyclability, targeting behaviors, adsorption or encapsulation processes, bioavailability, biocompatibility, cytotoxicity, and histopathological assessments, among others [40]. Herein, the drug delivery and cancer therapy applications of nanosponge-based systems (mostly, cyclodextrin-based, DNAzyme, and ethylcellulose nanosponges) are deliberated, with a focus on the crucial challenges and future directions.

## 2. Drug Delivery and Cancer Therapy

### 2.1. Cyclodextrin-Based Nanosponges

**Figure 2:**
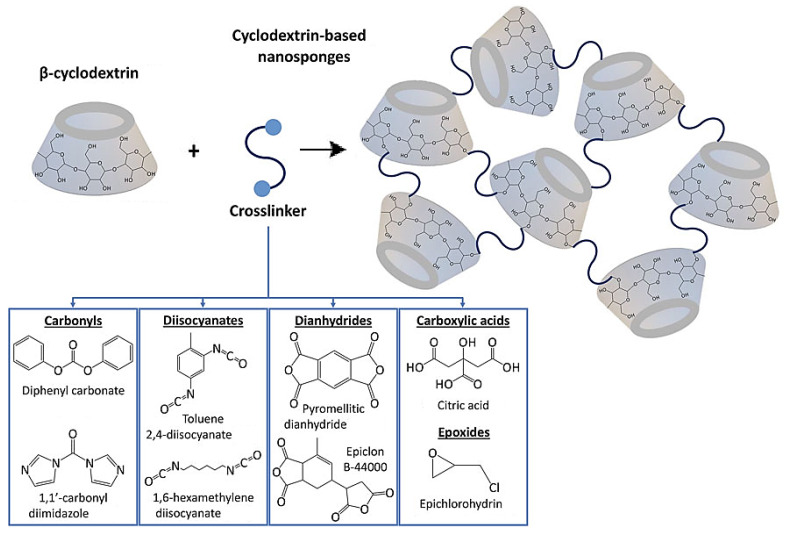
Assorted cross-linkers applied for manufacturing cyclodextrin nanosponge-based systems. Adapted from [12] with permission. Copyright 2022 Frontiers (CC BY 4.0).

Cyclodextrin nanosponges with unique properties, such as biocompatibility, porous structures, controlled-release behavior, and enhanced oral bioavailability, have been introduced as safe carriers of drugs/therapeutic agents for the treatment of various diseases (especially cancers/tumors) [14,41]. Although numerous in vitro cellular studies have been reported so far, further explorations ought to be focused on in vivo evaluations of these nanosponges (Table 1) [42]. Cyclodextrins have shown significant reactivity and can be directly co-polymerized with other monomers or grafted onto organic/inorganic compounds due to the presence of hydroxyl groups with the capability of a substitution/elimination process [43]. Cyclodextrin nanosponges with varying lipophilic cavities and a hydrophilic network based on the nature of cross-linkers can be considered as ideal alternates to improve the stability of compounds (e.g., volatile compounds) and the solubility of drugs/therapeutic agents (Figure 2) [44]. Notably, the porosity and surface area of nanosponges can be affected by the amount of cross-linkers; typically, with an increase in the amount of cross-linker usage, nanosponges with smaller sizes and greater porosity can be obtained. These nanosystems are resistant to organic solvents and can show good thermal stability (up to 300 °C), which makes them attractive candidates for a variety of nanoformulations [12]. For instance, cyclodextrin-based nanosponges have been designed to enhance the aqueous solubility of kynurenic acid as an antioxidant with therapeutic activities. Accordingly, the solubility of this antioxidant was highly enhanced, and higher drug-loading (~19.06%) and encapsulation proficiency (~95.31%) could also be obtained [45]. In addition, hyper-branched cyclodextrin-based nanosponges with high encapsulation efficiency (~80%) were developed for improving the physicochemical properties of norfloxacin (an antibiotic), thus facilitating its oral absorption; improved antimicrobial activity in sepsis models (in vivo) was discerned [46].

**Figure 3:**
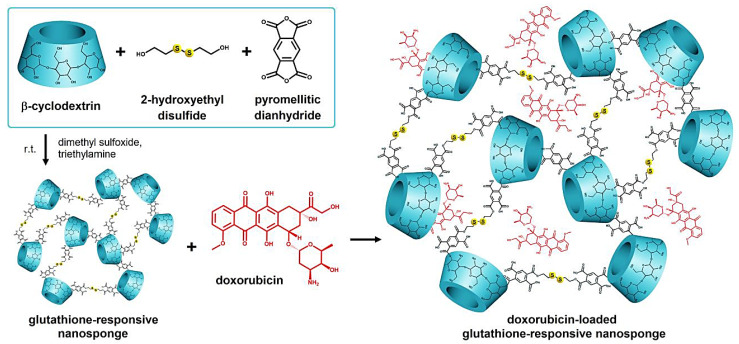
Preparative process for doxorubicin-loaded glutathione-responsive nanosponges with antitumor effects. Adapted from [60] with permission. Copyright 2020 Elsevier.

Glutathione-responsive cyclodextrin nanosponges have been designed for the targeted drug delivery of doxorubicin with improved anti-tumoral activity (in vitro and in vivo) (Figure 3). These nanosystems with good biosafety exhibited reduced drug resistance properties as they could be taken up via the active mechanisms and circumvent the efflux drug pump [60]. Similarly, glutathione bio-responsive nanosponges with high degradability, pH-responsive behavior, and efficient drug-loading capacity (~22.6%) were fabricated utilizing *β*-cyclodextrin-appended hyper-crosslinked polymer via the oligomerization of acryloyl-6-ethylenediamine-6-deoxy-*β*-cyclodextrin, acrylic acid, with *N,N*-bis (acryloyl)-cystamine (as a cross-linker) for the targeted delivery of doxorubicin. As a result, the release of doxorubicin was highly enhanced (~77.0%) in acidic (pH = 5.0) and cytosolic reducing (10 mM glutathione) environments, offering promising platforms for the targeted drug transport in tumor therapy [61].

Hyper-crosslinked cyclodextrin nanosponges (~316.4 ± 8.5 nm) synthesized via a solvent evaporation technique were loaded with artemether and lumefantrine (antimalarial agents) to improve their solubility and to acquire a controlled-release profile. In vitro evaluations illustrated the controlled-release behaviors of these nanosponges for 24 h; they exhibited good stability at 40°C for 3 months [62]. Additionally, *β*-cyclodextrin nanosponges were designed for transferring lipophilic drugs (e.g., dexibuprofen) and providing efficient delivery systems for improving the drug solubility [63]. Nanosponges were also constructed for improving the solubility of docetaxel in aqueous media with targeted delivery benefits [30,64,65]. Palminteri et al. [66] constructed a novel system using cyclodextrin-centered nanosponges for transferring the glutathione-mediated transport of resveratrol into the targeted cancerous cells. In addition, the oral bioavailability of avanafil and dapoxetin was improved by applying cyclodextrin-based nanosponges [67]. Magnetic nanosponges have shown great potential for targeted drug delivery as well [68]. These nanosystems were prepared after the addition of magnetite nanomaterial to the polymers of cyclodextrin and maltodextrin, crosslinked with 1,1′-carbonyldiimidazole. One study reported the design of magnetic nanosponges for the targeted delivery of doxorubicin with the loading capacity of ~3 wt%, wherein the loaded anticancer drug could be released with sustained kinetics over a prolonged period of time [68].

### 2.2. DNAzyme Nanosponges

**Figure 4:**
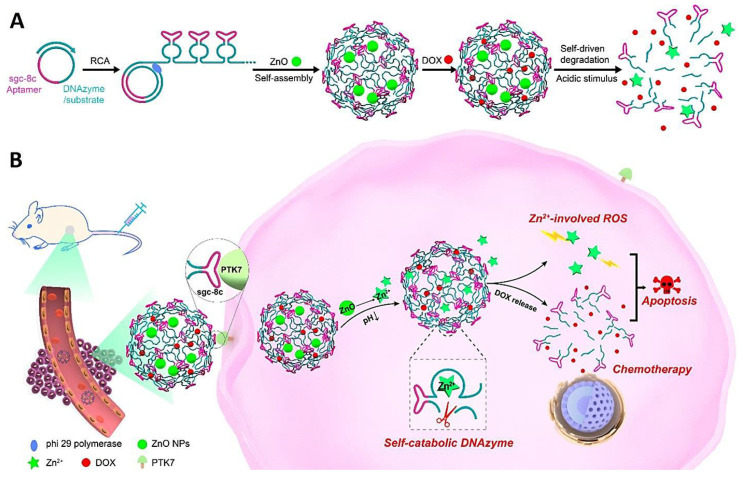
(**A**) Isothermal rolling circle amplification (RCA)-based assembly and acid-stimulated disassembly of nanosponge-based delivery systems containing doxorubicin (DOX), DNAzyme, and ZnO nanomaterials. (**B**) The intracellular dissolution of ZnO into Zn^2+^ ions can induce the formation of ROS and activate the cleavage of DNAzyme along with the stimulated release of the anticancer drug after specific uptake by cells. Adapted from [71] with permission. Copyright 2019 American Chemical Society.

The developments in bioinspired, self-catabolic DNAzyme nanosponges with controllable drug delivery behaviors and suitable gene silencing functions have been reported, thereby opening a new window in designing smart nanosystems with gene therapeutic and personalized biomedical commitments [69]. For instance, bioinspired self-catabolic DNAzyme nanosponges were fabricated with multifunctionality for controllable and targeted drug delivery and gene silencing with high efficiency [70]. Wang et al. [71] developed a DNAzyme-driven drug transport system containing the rolling circle oligomerized DNAzyme-substrate frameworks and the captured pH-receptive ZnO nanomaterials (Figure 4). The designed DNAzyme nanosponges could be encoded with multivalent tandem aptamer arrangements for targeted delivery into cancerous cells. Accordingly, the dissolution of ZnO into Zn^2+^ ions was stimulated by the acidic endo/lysosomal microenvironment to perform as co-factors of DNAzyme and the creators of therapeutic reactive oxygen species (ROS) [71].

**Figure 5:**
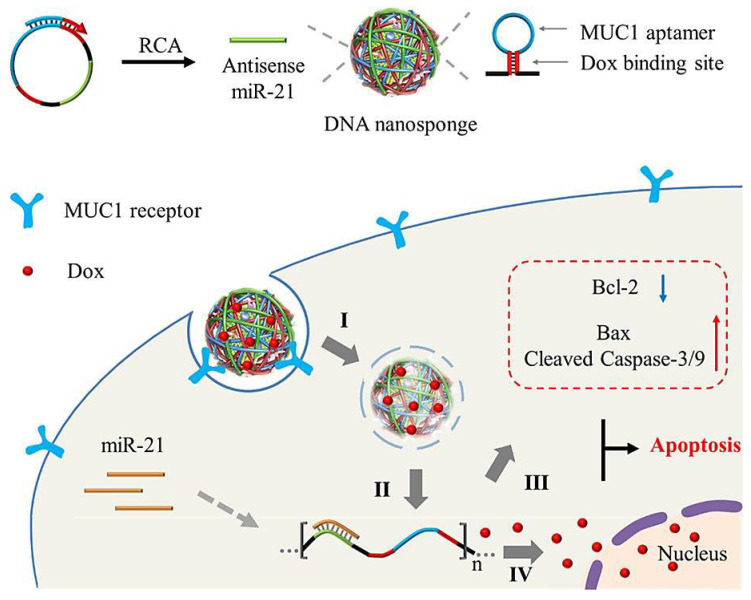
DNA nanosponges created for the clearance and adsorption of intracellular miRNA-21 (miR-21), along with regulatory effects of gene expression in tumor cells for synergistic and specific antitumor chemotherapy (I-IV). RCA: rolling circle amplification; Dox: doxorubicin. Adapted from [72] with permission. Copyright 2019 American Chemical Society.

Self-assembled DNA nanosponges were designed with multivalent tumor cell-fastening ligands to enable the tumor-explicit drug release with high efficacy (Figure 5) [72]. These nanosponges were applied for adsorption and clearance of intracellular miRNA-21 and could be damaged under acidic pH conditions in endo/lysosomes to provide plentiful miRNA-21 binding sites and induce the discharge of doxorubicin. They triggered synergistic antitumor chemotherapy, which ensued as a result of the co-delivery of doxorubicin and antisense oligonucleotides for miRNA-21. The improvement of antitumor chemotherapy by DNA nanosponges could be attained via the modification of the cell apoptosis-associated gene expression triggered by them [72].

**Figure 6:**
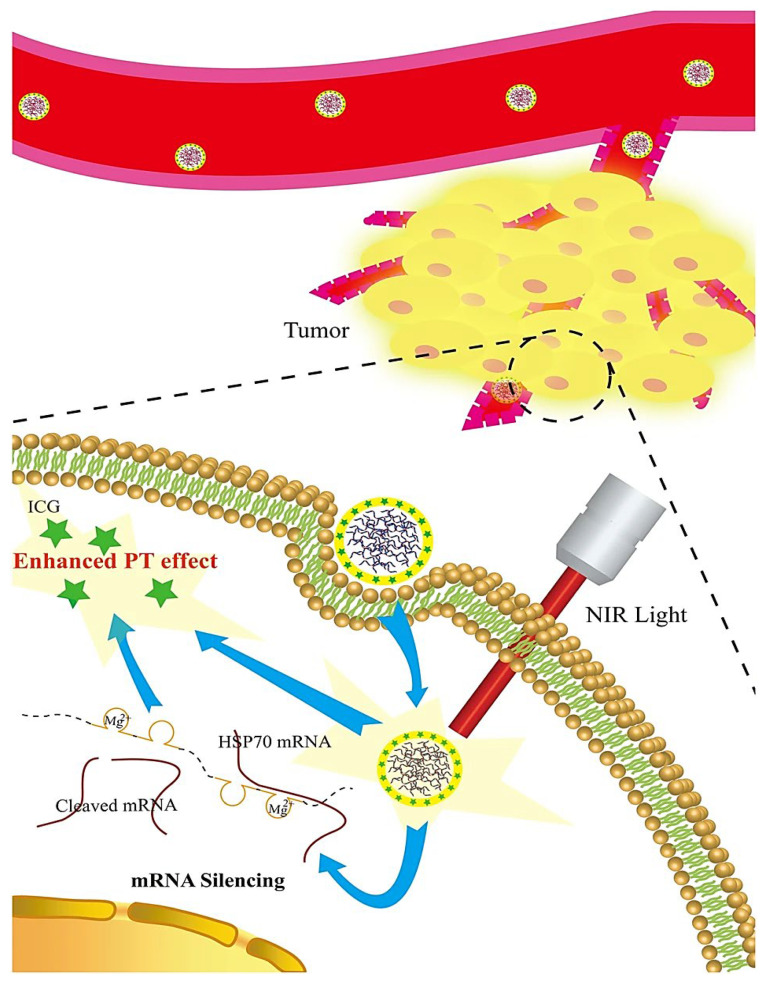
DNAzyme-based nanosponges applied for targeted photothermal therapy of tumors with multimodal imaging potentials. PT: photothermal; NIR: near-infrared; ICG: indocyanine green (photothermal small molecule). Adapted from [69] with permission. Copyright 2018 Springer Nature (CC BY 4.0).

Dynamic DNA nanosponges with appropriate stability and biocompatibility were designed for DNAzyme-mediated gene regulation and programmable tumor-targeted delivery with high efficiency [73]. After environmental stimulation, the performance of DNAzyme was increased and the cleavage of RNA was accelerated by a supplementary catalytic co-factor. Notably, the generation of O_2_ and ^1^O_2_ was accelerated as a supplementary treatment, providing concurrently self-enhanced gene-photodynamic cancer therapy [73]. Future studies ought to focus on the clinical translation of these oligonucleotide-based drugs for cancer therapy. Sponge-like nanoplatforms were developed via the simple assembly of a cationic polymer and a long single strand of DNA encoded with sequences of multivalent deoxyribozyme (DNAzyme) (Figure 6) [69]. These nanosponges were employed for the photothermal therapy of cancers to overcome thermal resistance. The DNAzymes catalytically cleaved the HSP70 mRNAs and downregulated the expression of the subsequent proteins to obtain protection effects towards the cancer cells (MCF-7) from destruction by hyperthermia, sensitizing these cancerous cells to heat via the overexpression inhibition of HSP70. These nanosponges could be applied for multimodal imaging due to their efficient tumoral accumulation capabilities with an enhanced permeability and retention effect [69].

### 2.3. Ethylcellulose Nanosponges

Ethylcellulose nanosponges were constructed via an ultrasonic-assisted emulsion solvent evaporation method for the targeted delivery of withaferin-A with anticancer properties [74]. Accordingly, the examined drug was successfully entrapped (~85 ± 11%) into the nanosponges (~117 ± 4 nm) for anticancer activity against MCF-7 cells (the half-maximal inhibitory concentration = ~1.57 ± 0.091 µM). The possible mechanism for the elimination of cancer cells was based on apoptosis. After the in vivo evaluation of the nanosponge-based system, the results were consistent with those obtained with cisplatin [74]. In addition, to improve the bioavailability of Abemaciclib (an anticancer drug against breast cancer), ethylcellulose nanosponges were created with sustained-release behavior via an emulsion solvent diffusion technique [75]. Consequently, the nanosponges exhibited high stability and sustained release of the drug (~77.12 ± 2.54%) in 24 h, with efficient cytotoxic activity versus MCF-7 and MDA-MB-231 human breast cancerous cells [75]. Spherical ethylcellulose nanosponges with sustained-release behavior were prepared via a quasi-emulsion solvent diffusion technique and could be deployed for the delivery of hesperetin with anti-carcinogenic, tumor necrosis, and antioxidant effects [76]. These nanosponges could retard the drug discharge (~39.98%) for up to 8 h relative to the neat drug (~70.74%) and the physical blend (~73.72%), with robust downregulating influences on cytokines [76].

Almutairy et al. [77] developed ethylcellulose nanosponges for improving the oral bioavailability of olmesartan medoxomil with antihypertensive potentials (in vivo). This nanosystem with sustained-release behavior exhibited higher cytotoxicity against A549 lung cell lines in relation to the neat drug and, additionally, could significantly reduce the systolic blood pressure compared to the control and pure drug [77]. In addition, lemongrass-loaded ethylcellulose nanosponges were formulated via an emulsion solvent evaporation method, which displayed enhanced in vivo antifungal activity against *Candida albicans* strain ATC 100,231 and reduced irritation [78]. Ethylcellulose nanosponges (~272.92 ± 12.31 nm) fabricated by the double emulsion solvent evaporation technique exhibited the entrapment efficiency for carboplatin of ~56.27 ± 2.52%. The designed nanosponges had sustained drug releases of 79.03% (pH = 4.5) and 95.94% (pH = 6.8) within 12 h, making them efficient nanocarriers with the sustained release of hydrophilic therapeutic agents [79].

Nanosponges have been synthesized through an emulsion solvent evaporation technique deploying polyvinyl alcohol (PVA) and ethylcellulose for the targeted delivery of ribociclib, a kinase inhibitor against metastatic breast cancer [80]. Consequently, the encapsulation of the drug was successfully accomplished within the porous polymeric matrix. After in vitro analyses, it was revealed that the drug release was highly accelerated, with a maximum drug release through the ribociclib-loaded nanosponges (~81.85 ± 0.37%). This nanosystem exhibited high cytotoxic effects against MDA-MB-231 and MCF-7 breast cancerous cell lines, with a higher degree of apoptosis compared to the free ribociclib, offering promising platforms for targeted drug delivery against metastatic cancers, with a higher safety and efficacy profile [80]. Additionally, nanosponge-based platforms (~261.0 ± 3.5 nm) endowed with a sustained mode were constructed from ethylcellulose and PVA via an emulsion solvent evaporation method for targeted delivery of brigatinib as tyrosine kinase inhibitors against lung cancer cells [81]; the entrapment efficiency was ~85.69 ± 0.04% and the drug loading was ~17.69 ± 0.01%. The nanosystems exhibited sustained drug release (~86.91 ± 2.12%) for ~12 h, thereby efficiently reducing the cell viability of the human lung cancer cell line A549 [81].

## 3. Conclusions and Future Perspectives

Nanosponge-based systems endowed with remarkable porosity, simple functionalization processes, unique architectures, eco-friendliness, and cost-effectiveness have been explored as promising alternatives to targeted drug delivery and cancer therapy. Among them, cyclodextrin nanosponges exhibited unique properties, high biocompatibility, negligible toxicity, and ease of surface modifications, which renders them the predominantly evaluated nanosponges in bio- and nanomedicine. Future studies ought to be directed towards the efficient functionalization of nanosponges for the reduction in possible toxicity, the improvement of their biosafety, and the enhancement of their specificity/selectivity. These structures can be applied to improve the solubility of the drugs/therapeutic agents and protect them from degradation; by changing the concentration of polymers/other materials and the cross-linker ratio, innovative nanosponges can be obtained endowed with multifunctionality and different properties. Notably, additional explorations need to focus on their biodistribution and biocompatibility. To better improve the physicochemical properties and functionality of nanosponges, further optimization studies are still necessary; their complexation performances, structural variations, commercialization, long-term biosafety analyses, low-cost/large-scale production, and specific nanotoxicological assessments ought to be comprehensively focused on in future explorations. Additional studies are warranted to focus on the specific surface functionalization or modification of nanosponges using distinct materials, such as fluorescent compounds, magnetite nanoparticles, folic acid, etc., to fabricate multifunctional systems with cancer/tumor theranostic applications.

## Figures and Tables

**Table 1:** Some selected examples of nanosponges in biomedicine with attractive benefits.

Nanosponge-Based Systems	Applications	Advantages/Properties	Refs.
Cyclic nigerosyl-1,6-nigerose-based nanosponges	Drug delivery	- Controlled and sustained release of doxorubicin - High biocompatibility - pH-sensitive release	[47]
Cyclodextrin nanosponges	Drug delivery (erlotinib)	- Improved dissolution rate and efficiency - Enhanced oral bioavailability - Increased solubility	[48]
Cyclodextrin nanosponges	Drug delivery (camptothecin); tumor therapy	- Prolonged release of the drug - Enhanced anti-proliferative function and DNA damage capabilities for cancer cells (in vitro) - Improved solubility	[49]
Cyclodextrin nanosponges	Drug delivery (paclitaxel); cancer therapy	- Improved oral bioavailability - Reduced toxicity	[50]
Cyclodextrin nanosponges	Drug delivery (flutamide)	- Improved dissolution rate - Good biocompatibility; non-toxicity (in vitro) - Improved solubility	[51]
Cyclodextrin nanosponges	Drug delivery (camptothecin); cancer therapy	- Increased solubility - High inhibitory effects against the growth, the metastasization, and the vascularization of orthotopic anaplastic carcinoma of the thyroid xenografts (in vivo) - No noticeable toxic effects (in vivo)	[52]
Cyclodextrin nanosponges	Anticancer drug delivery (curcumin); cancer therapy	- Increased dissolution - Enhanced photostability of curcumin - Increased toxicity to cancer cell lines (MCF-7 cells)	[53]
Cyclodextrin nanosponges	Drug delivery (nifedipine)	- Improved oral bioavailability of drug - Enhanced stability in normal or stress conditions - Sustained release behavior	[54]
Cyclodextrin nanosponges	Targeted delivery of the anti-restenotic agent, DB103	- The biphasic drug release - Sustained release behavior (gradual and steady drug discharge) - Improved bioavailability	[15]
Fluorescent hyper-crosslinked *β*-cyclodextrin-carbon quantum dot hybrid nanosponges	Anticancer drug delivery (doxorubicin)	- High biocompatibility - Strong bright blue fluorescence - High photoluminescence quantum yield of ~38.0% - pH-responsive controlled release - High efficiency	[55]
Cyclodextrin nanosponges	Drug delivery system (dithranol delivery for psoriasis)	- Improved solubility - Enhanced stability and photostability - High cytocompatibility - Improved in vitro antioxidant and in vitro inflammatory activity	[56]
Cyclodextrin nanosponges	Drug delivery formulation (griseofulvin)	- High drug-loading capacity - High entrapment efficiency - A suitable pathway for masking the bitter taste of griseofulvin and enhancing its oral bioavailability	[57]
Cyclodextrin nanosponges	Drug formulation (acetyl salicylic acid, ASA)	- Prolonged drug release - High encapsulation efficiency - Accelerated stability	[58]
Cyclodextrin nanosponges	Drug delivery system (telmisartan)	- Improved bioavailability - Increased solubility - Synergistic enhancement of drug dissolution via the modulation of micro-environmental pH and modification of drug amorphization	[59]

## Data Availability

Not applicable.
